# Explainability and Human Oversight for AI-Generated Exercise Guidance in Digital Healthcare: A Governance-Oriented Narrative Review

**DOI:** 10.3390/healthcare14121716

**Published:** 2026-06-15

**Authors:** Kaijiang Pan, Caihua Huang, Xinyu Lin, Shengqi Huang

**Affiliations:** 1 School of Marxism, Xiamen Ocean Vocational College, Xiamen 361100, China; kj-pan@foxmail.com; 2 Research and Communication Center for Exercise and Health, Xiamen University of Technology, Xiamen 361024, China; shengqi.huang@foxmail.com; 3 School of Film and Communication, Xiamen University of Technology, Xiamen 361024, China; xinyulinxmut@outlook.com

**Keywords:** artificial intelligence, generative AI, digital healthcare, exercise prescription, explainable artificial intelligence, patient safety, human oversight, telerehabilitation

## Abstract

**Background:** Large language models and other generative artificial intelligence (AI) tools are increasingly being embedded in digital healthcare services, including mobile health applications, telerehabilitation, remote monitoring, and hybrid care pathways. In this review, digital healthcare refers to technology-mediated healthcare services in which digital platforms, mobile applications, wearables, remote communication, and AI-enabled interfaces support health assessment, self-management, rehabilitation, clinical decision support, or service delivery. When AI-generated exercise guidance moves from general education to individualized recommendations about dose, progression, contraindications, or rehabilitation, it may become directly actionable and safety-relevant. **Objectives**: This review aimed to clarify when AI-generated exercise guidance in digital healthcare may warrant safety-relevant governance attention and to outline implementation considerations for explainability, human oversight, and service-level governance. It addresses a gap in the literature: general AI-governance and exercise-prescription discussions rarely specify how point-of-use explanations, review thresholds, and escalation safeguards can be organized for directly actionable AI exercise guidance. **Methods**: We conducted a governance-oriented narrative review of peer-reviewed literature and representative regulatory or guidance documents. This review was not designed as a systematic review, scoping review, or exhaustive evidence map; transparent source mapping was used to support conceptual synthesis. Searches and source mapping focused on generative AI, large language models, explainable AI, clinical decision support, digital health, mobile health, exercise prescription, rehabilitation, trust, automation bias, and human oversight. Sources were included when they informed the safety, explainability, governance, or real-world implementation of patient-facing AI-generated exercise guidance. Extracted material was grouped by evidentiary role and synthesized through framework synthesis and governance mapping to distinguish literature-supported observations, author interpretation, and proposed implementation tools. Results: The included sources were first organized into five thematic groups: digital exercise delivery and exercise-prescription evidence; explainability, trust, and automation bias literature; professional responsibility, ethics, and patient disclosure literature; regulatory and policy documents; and digital literacy, patient/clinician attitudes, and equity literature. The synthesis then proceeded from safety relevance to explanation needs, human oversight and escalation needs, and selected regulatory and policy signals before translating these strands into conceptual and implementation-oriented outputs rather than empirically validated instruments. AI-generated exercise guidance was most safety-relevant in scenarios involving individualized dose, progression, contraindication-sensitive action, or rehabilitation strategy. Across the included sources, generic transparency alone was not sufficient to support reviewable use; relevant explanation elements included evidence sources, risk warnings, reasoning paths, and reasonable alternatives. Oversight considerations varied with embodied risk, clinical ambiguity, user vulnerability, and likelihood of direct enactment. Implementation considerations linked interface design, clinical review, escalation, auditability, and post-deployment monitoring. Conclusions: AI-generated exercise guidance in digital healthcare may warrant governance attention as a patient-safety and accountability issue when it influences actionable exercise decisions. The proposed framework offers a conceptual basis for designing more reviewable and accountable mobile and remote exercise-support services. Future work can validate these outputs in patient-facing services, clinician review workflows, usability studies, implementation pilots, and safety evaluations.

## 1. Introduction

Large language models (LLMs) and other generative AI systems are increasingly being embedded in interfaces that support exercise counseling, remote rehabilitation, app-based self-management, and hybrid models of care [1,2]. In these settings, AI-generated outputs may extend beyond education or motivation and begin to shape individualized recommendations about exercise type, intensity, frequency, progression, warning symptoms, and risk management [3,4,5].

Digital healthcare is used in this article as an umbrella term for technology-mediated healthcare services in which digital platforms, mobile applications, wearable devices, remote communication, and AI-enabled interfaces support assessment, self-management, rehabilitation, clinical decision support, or service delivery. This definition is broader than mobile health alone and narrower than all consumer wellness technology because it includes care-related decisions, professional review pathways, or health-service accountability.

This distinction matters because exercise guidance is often enacted directly. A recommendation about intensity, progression, stop rules, or contraindicated activity can quickly translate into bodily exertion, symptom change, or recovery-related consequences. For older adults, people living with chronic disease, and patients in rehabilitation, small errors in dose or progression may carry clinical significance [6,7,8,9].

The central question is therefore not merely whether AI-generated output sounds plausible, but whether it is sufficiently explainable, reviewable, and governable for patient-facing use. In this review, explainability refers to the extent to which patients, clinicians, exercise professionals, and service organizations can understand the basis, key inputs, evidence sources, safety assumptions, limitations, and alternatives behind an AI-generated recommendation well enough to use, review, modify, or reject it responsibly. FITT-VP refers to frequency, intensity, time, type, volume, and progression, which are commonly used dimensions for structuring exercise prescription. The review focuses on the point at which such guidance becomes safety-relevant, the types of explanation needed by users and professionals, and the oversight arrangements required for responsible implementation.

Previous work on explainable AI in healthcare has clarified the importance of transparency, trust, professional accountability, and governance [10,11,12,13,14,15,16,17,18,19,20]. However, exercise guidance presents a distinctive implementation problem: it is embodied, frequently self-enacted, often delivered remotely, and commonly presented in language that users can follow immediately. This creates a gap between general discussions of explainable AI and the practical needs of exercise-related digital healthcare services.

Accordingly, this review addresses the following question: What forms of explainability and human oversight are most relevant when AI-generated exercise guidance in digital healthcare moves from general wellness support toward individualized, directly actionable, or clinically consequential guidance?

To clarify its disciplinary scope, this article is positioned as a governance-oriented narrative review of digital healthcare focused on exercise-related patient safety, rather than as a systematic review, scoping review, comprehensive evidence map, comprehensive exercise science review, or general AI ethics paper.

## 2. Materials and Methods

This article was designed as a governance-oriented narrative review rather than a systematic review, scoping review, comprehensive evidence map, or meta-analysis. The aim was to integrate heterogeneous literature from digital health, exercise prescription, rehabilitation, explainable AI, clinical decision support, ethics, and regulation in order to develop a conceptual and implementation-oriented framework for AI-generated exercise guidance. Rather than estimating intervention effects, mapping all available evidence, or presenting validated empirical instruments, the review used narrative source integration to clarify safety-relevant use cases, explanation needs, oversight functions, and implementation-governance considerations. The review explicitly separates literature-supported observations, author interpretation, and proposed implementation tools.

Searches were conducted initially on 10 January 2026 and updated on 15 May 2026 across PubMed/MEDLINE, Google Scholar, and official regulatory or guidance websites from China, the European Union, the World Health Organization, and the United States [21,22,23,24,25,26]. During the present revision, a targeted journal-specific check of Healthcare was also conducted on 29 May 2026 to identify recent articles directly relevant to AI exercise prescription, patient-centered digital health decision support, rehabilitation, explainability, and equity. The PubMed/MEDLINE search used the following string: (“large language model” OR “generative AI” OR “artificial intelligence” OR “explainable AI” OR “clinical decision support”) AND (“exercise prescription” OR “exercise guidance” OR “physical activity” OR “rehabilitation” OR “telerehabilitation” OR “digital health” OR “mobile health”) AND (“patient safety” OR “human oversight” OR “automation bias” OR “trust” OR “governance”). Google Scholar was used as a supplementary source for citation tracking and interdisciplinary coverage rather than as the sole basis for inclusion; for transparency of narrative source selection, screening was limited to the first 100 relevance-ranked results for each phrase combination after duplicate, inaccessible, and clearly irrelevant records were removed. Google Scholar searches used equivalent phrase combinations, including “large language models” AND “exercise prescription”, “generative AI” AND “exercise guidance”, “explainable AI” AND healthcare AND “human oversight”, “clinical decision support” AND “automation bias”, and “telerehabilitation” AND “digital health” AND exercise. Official websites were searched using combinations of “artificial intelligence”, “generative AI”, “clinical decision support”, “medical device”, “transparency”, “human oversight”, “software”, “change control”, and “governance”.

Eligible sources were English-language peer-reviewed articles, consensus or review articles, empirical studies, ethical analyses, and official guidance documents published mainly from 2019 onward, with earlier foundational ethical sources included when necessary [27]. Sources were selected when they directly informed at least one of four operational domains: (1) safety-relevant exercise prescription or digital exercise delivery; (2) AI explainability, trust, automation bias, or reviewability in healthcare; (3) human oversight, professional accountability, or governance of AI-enabled healthcare; and (4) regulatory or policy requirements relevant to patient-facing or clinician-facing AI guidance.

To strengthen the empirical grounding of the synthesis, source selection deliberately included empirical mHealth exercise-intervention studies, systematic reviews of app-based exercise prescription, telerehabilitation or remote rehabilitation sources, digital health literacy studies, patient-attitude research, and recent Healthcare articles on AI exercise prescription, patient-centered digital health decision support, disability-related rehabilitation, and implementation equity alongside conceptual AI governance literature [28,29,30,31,32,33,34,35,36,37].

Sources were excluded when they focused solely on technical model performance without healthcare implementation relevance, addressed diagnostic imaging without transferable implications for patient-facing decision support, discussed general wellness applications without implications for exercise dose, contraindications, rehabilitation, or safety monitoring, or consisted of non-authoritative commentary without a clear evidentiary or policy basis. Because the purpose was narrative and conceptual synthesis, PRISMA-style reporting, formal risk-of-bias scoring, and meta-analysis were not performed.

Because the included materials combined empirical studies, reviews, conceptual ethics papers, and official governance documents, a single quantitative quality-appraisal tool was not appropriate. Instead, each source was categorized by evidentiary role before synthesis: empirical studies and systematic reviews informed implementation and user-facing safety considerations; exercise-prescription and rehabilitation sources informed clinical plausibility; official regulatory or policy documents informed governance signals; and conceptual ethics or explainability papers informed interpretive concepts. This source-role categorization provided the basis for later framework construction and avoided treating all materials as equivalent evidence in the narrative synthesis.

Screening and charting were conducted by two authors (K.P. and X.L.), with disagreements discussed with C.H. until consensus was reached. For each included source, the extracted data items were source type, population or user group, clinical or service setting, AI system or digital health technology, exercise or rehabilitation context, safety concern, explainability mechanism, human oversight mechanism, governance implication, and relevance to patient-facing digital healthcare. The synthesis then proceeded through four traceable steps: first, charting source statements related to safety relevance, explanation, oversight, and governance; second, grouping recurrent concerns by evidentiary role and implementation context; third, mapping those concerns to digital healthcare workflow points such as point-of-use explanation, professional review, escalation, auditability, and monitoring; and fourth, after the literature-supported themes and selected policy signals had been synthesized, translating the mapped considerations into the proposed risk-gradient framework and implementation tools as proposed implementation tools rather than validated instruments. For the current revision, all bibliographic records were rebuilt from DOI-, PubMed-, publisher-, or official-source records; mismatched author, title, journal, year, volume, page, and DOI fields were corrected, and in-text citation placement was rechecked.

## 3. Results and Synthesis

### 3.1. Thematic Grouping of Included Sources

Before deriving implementation considerations, the 37 included sources were grouped by thematic and evidentiary role. This grouping was used to make the narrative synthesis traceable, to show which bodies of literature informed each part of the argument, and to avoid presenting the proposed framework as if it were derived from a single homogeneous evidence base. Some sources contributed to more than one theme; the grouping below therefore indicates primary roles in the synthesis rather than mutually exclusive evidence categories.

Table 1 summarizes the search, selection, data extraction, synthesis, evidence-role categorization, and reference-verification process.

Table 2 summarizes the thematic grouping of included sources and their primary roles in the narrative synthesis.

This thematic grouping guided the subsequent synthesis: exercise-prescription and rehabilitation sources were used to define safety-relevant use cases; explainability and automation-bias sources informed reviewable explanation; ethics and responsibility literature informed patient disclosure and professional oversight; regulatory documents were summarized as selected policy signals; and digital-literacy, attitudes, and equity sources informed implementation and access considerations.

### 3.2. Safety Relevance of AI-Generated Exercise Guidance

Exercise-prescription, rehabilitation, and digital exercise-delivery sources indicate that AI-generated exercise guidance becomes safety-relevant when it does more than encourage generally healthy behavior and instead shapes individualized dose, progression, exercise selection, warning thresholds, or rehabilitation strategy in ways that may alter bodily risk, symptom burden, or recovery trajectories [3,4,5,6,7,8,9,35]. In these situations, the output functions less like low-risk wellness content and more like patient-facing healthcare guidance.

Based on these sources, we interpret three conditions as especially important for identifying safety-relevant AI exercise guidance. First, the recommendation materially determines individualized dose or progression, such as the frequency, intensity, time, type, volume, and progression of exercise. Second, the recommendation governs contraindication-sensitive or rehabilitation-sensitive action, including decisions about pain, symptom monitoring, cardiovascular risk, musculoskeletal injury, frailty, chronic disease, palliative-care needs, or disability-related rehabilitation. Third, the guidance is likely to be enacted without meaningful prior professional reinterpretation, as commonly occurs in mobile and remote care pathways [3,4,5,6,7,8,9,35,37].

When these conditions are present, explainability and human oversight function as practical mechanisms for patient safety, informed use, professional review, and organizational accountability, a point supported by automation-bias, disclosure, responsibility, and AI exercise-prescription sources [14,15,16,20,31,32,33,35].

### 3.3. Explanation Needs Identified Across Sources

Across the explainability, trust, automation-bias, and patient-centered digital health sources, explainability was most useful when treated as a reviewable service function, not only as a technical property [10,11,12,13,14,15,17,19,36]. These sources suggest that transparency supports safe reliance only when users and professionals can inspect why a recommendation was generated, what evidence or data shaped it, what assumptions or uncertainties remain, and what alternatives are available. Patient-centered digital health work similarly highlights stakeholder demand for AI transparency, clinician oversight, and accessible explanations [36]. Disability-focused research further indicates that explainability can support accessible, personalized rehabilitation rather than assuming a single user profile [37]. The practical question was therefore whether patients, clinicians, exercise professionals, and organizations could understand the basis, safety assumptions, and practical consequences of a recommendation well enough to use, review, modify, or reject it.

### 3.4. Human Oversight and Escalation Needs

Across the professional responsibility, ethics, patient-disclosure, institutional trustworthiness, and exercise-AI ethics literature, human oversight emerged as a proportionate care-pathway function rather than as a general aspiration [13,16,18,20,31,32,33,35]. Services may define three levels of response before deployment: recommendations that can be released with standardized safeguards, recommendations that warrant clinician or exercise-professional confirmation, and recommendations that trigger escalation or referral.

This proportional approach is especially relevant in mobile, remote, rehabilitation, and palliative-care contexts, where users may act on personalized exercise advice within minutes [3,4,5,28,29,30,35]. In disability-related contexts, safety, dignity, accessibility, and digital-literacy needs may vary substantially, reinforcing the value of inclusive escalation and review pathways [37]. Clear review thresholds and escalation criteria can reduce the gap between AI output and safe implementation and help determine when a recommendation can be released, reviewed, modified, suspended, or referred for additional care.

Oversight also protects clinical judgment and patient autonomy. Clinicians and exercise professionals may benefit from the ability to question, revise, or reject AI-generated recommendations when risk, uncertainty, inconsistency, or user vulnerability is high, and patient-facing services can use disclosure practices that clarify when AI is being used and whether professional review is available [16,20,31,32,33,35].

### 3.5. Selected Regulatory and Policy Signals Relevant to Implementation Governance

After safety relevance, explanation needs, and oversight or escalation needs were synthesized, official regulatory and policy sources were reviewed as selected signals relevant to implementation governance rather than as an a priori jurisdictional framework. Across China, the European Union, and the United States, the regulatory and ethical significance of AI-enabled guidance is shaped by intended use, user population, risk, and the ability of professionals or users to understand and review the basis of a recommendation. Table 3 summarizes selected regulatory and policy signals relevant to AI-generated exercise guidance in digital healthcare.

The following regulatory and policy signals are used as implementation-oriented considerations derived from the narrative synthesis, not as validated measurement items. They are intended to support service design, review, and future evaluation rather than to replace local clinical judgment or regulatory classification.

These selected policy signals were therefore used to inform implementation-governance questions, not to function as a predefined comparative framework. Implementation teams may specify whether an AI exercise function could qualify as medical-device software or clinical decision support. They can also define patient disclosures, review triggers, retained audit trails, update documentation, and procedures for reviewing adverse events, unsafe recommendations, or near misses.

### 3.6. Translation into Proposed Implementation Tools

**Figure 1:**
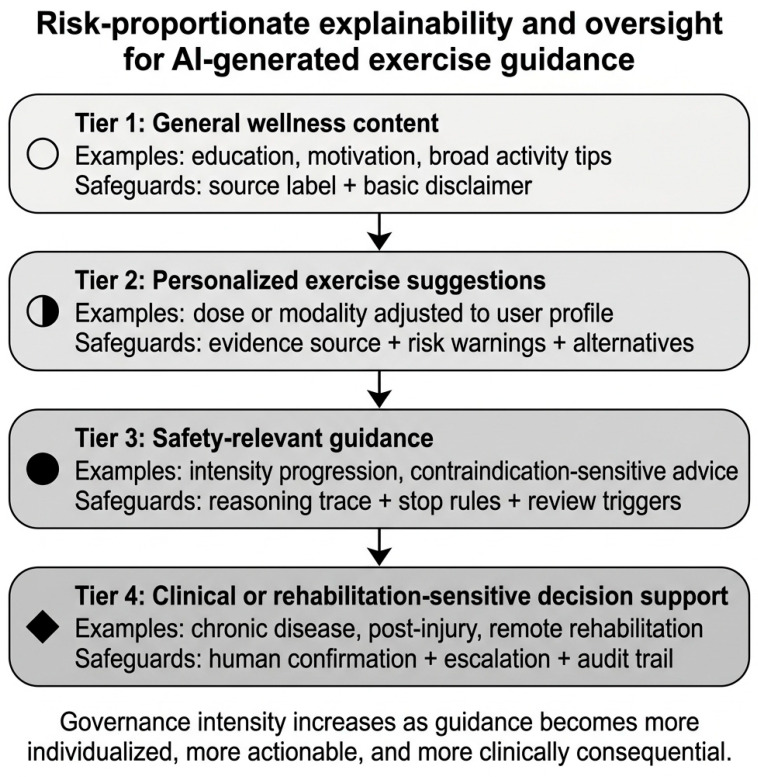
Proposed risk gradient for explainability and human oversight in AI-generated exercise guidance. The figure uses symbols, grayscale tiers, and textual safeguards to show that governance intensity increases as guidance becomes more individualized, actionable, and clinically consequential. It is proposed as a conceptual implementation aid rather than a validated framework, checklist, or regulatory classification tool.

After the literature-supported observations were synthesized, we interpreted them as suitable for translation into proposed implementation tools rather than validated instruments. The outputs include explanation elements for point-of-use review (Table 4), a risk-proportionate framework (Figure 1), and a simplified implementation checklist (Table 5). The explanation elements in Table 4 operationalize four functions identified across the synthesis: evidence-source disclosure, risk-warning communication, reasoning-path clarification, and presentation of reasonable alternatives [10,11,12,13,14,15,16,17,19,20,23,24,25,26,36].

The following elements are proposed as implementation-oriented considerations derived from the narrative synthesis, not as validated measurement items. They are intended to support service design, review, and future evaluation rather than to replace local clinical judgment or regulatory classification.

## 4. Discussion

### 4.1. Proposed Framework

Building on Section 3, the proposed framework translates safety relevance, explanation needs, oversight and escalation needs, and selected regulatory or policy signals into implementation-oriented tools. Figure 1 presents an implementation-oriented risk gradient that uses tier labels, symbols, and textual safeguards so that meaning does not depend on color alone. The framework is intended as a practical conceptual and implementation aid rather than a validated scoring tool, comprehensive evidence map, substitute for jurisdiction-specific regulatory classification, or replacement for clinical judgment.

The following risk-gradient framework is proposed as an implementation-oriented consideration derived from the narrative synthesis, not as a validated measurement instrument. It is intended to support service design, review, and future evaluation rather than to replace local clinical judgment or regulatory classification.

### 4.2. Applying the Framework in Digital Healthcare Services

At the lowest level, general wellness content may use scope statements, source labels, and disclaimers. Personalized exercise suggestions may add stronger evidence disclosure and risk warnings. Safety-relevant guidance may include reasoning traces, contraindication logic, stop rules, and review triggers. Clinical or rehabilitation-sensitive decision support is likely to warrant human confirmation, escalation pathways, audit trails, and post-deployment monitoring.

An illustrative scenario helps clarify this point.

Consider an older adult with hypertension using an app-based strength-training service [4,5,6,7,9]. If the system detects that resting blood pressure is above the service threshold on that day, a safe patient-facing response would not merely reduce intensity. It would also explain that the adjustment is linked to cardiovascular risk, recent measurements, and reduced physiological reserve; present a lower-intensity alternative; and direct the user to seek clinical review if warning symptoms are present [23,24].

## 5. Practical Implications

### 5.1. Implications for Developers

For developers, the reviewed explainability, automation-bias, regulatory, and patient-centered digital health sources indicate that explainability and safety communication can be built into the interface and model workflow from the outset [10,11,12,13,14,15,17,19,23,24,25,26,36]. Patient-facing systems may show evidence provenance, contraindication warnings, stop rules, and reasonable alternatives in language that supports real use. Auditability can be supported by retaining reviewable traces of retrieved sources, decision factors, version changes, human overrides, and escalation events where appropriate.

### 5.2. Implications for Clinicians and Exercise Professionals

For clinicians and exercise professionals, the synthesis supports interpretive engagement rather than deference. Ethics, disclosure, responsibility, and Healthcare exercise-AI sources indicate that professionals remain responsible for judging whether outputs fit the patient’s diagnosis, symptoms, tolerance, goals, dignity, and care context [13,16,20,31,32,33,35]. Interfaces can support this role by making recommendations easy to review, modify, or reject.

### 5.3. Implications for Healthcare Organizations and Regulators

For healthcare organizations, regulatory and policy documents emphasize that patient-facing AI guidance can be embedded in governance structures rather than treated only as isolated model output [20,21,22,23,24,25,26]. Based on these sources, services may define approved use cases, risk tiers, and review triggers before deployment. They may also specify escalation workflows, documentation practices, and responsibilities for monitoring incidents. Regulators and policymakers can support implementation by clarifying how risk-proportionate explanation, professional reviewability, and post-deployment monitoring apply to guidance that patients may enact directly.

### 5.4. Patient Disclosure, Informed Use, and Equity

Patient-disclosure, trust, and patient-centered digital health sources suggest that patient-facing AI exercise services can disclose, in plain language, whether guidance was generated or adapted by AI, which inputs shaped it, what evidence or protocol supports it, what uncertainty remains, what stop rules apply, and whether professional review is available [16,20,31,32,33,36]. Such disclosure can support informed use within the workflow rather than function only as a one-time consent form.

Global applicability is also shaped by lower-resource and differently regulated settings. Where formal AI pathways, specialist capacity, or monitoring infrastructure are limited, the framework may be translated into minimum safeguards: use-case triage, plain-language warnings, conservative defaults, accessible escalation routes, proportionate documentation, and local adaptation. Digital literacy, patient-attitude, health-equity, and disability-focused sources indicate that these safeguards are especially important for older adults, people living with chronic disease, people with disabilities, and users with limited digital literacy [28,29,30,34,37]. Interfaces can also provide accessible language, multimodal warnings, caregiver or professional support options, and non-digital alternatives where feasible.

### 5.5. Real-World Implementation and Evaluation

Implementation also warrants real-world evaluation. Digital exercise and AI exercise-prescription sources indicate that digital interventions can improve access and personalization, but safer deployment is likely to depend on adherence, monitoring, and professional workflow [3,4,5,35]. Patient-centered digital health, digital literacy, and equity sources further indicate that evaluation can examine user understanding, accessibility, and support for vulnerable users [28,29,30,34,36,37]. Evaluation can examine whether users understand recommendations, professionals can review them efficiently, escalation triggers work, and services reduce rather than widen disparities in safe use.

As a final implementation-oriented output, Table 5 provides a simplified implementation checklist linking framework elements to governance questions, minimum safeguards, and example actions.

The following checklist items are proposed as implementation-oriented considerations derived from the narrative synthesis, not as validated measurement items. They are intended to support service design, review, and future evaluation rather than to replace local clinical judgment or regulatory classification.

## 6. Strengths, Limitations, and Future Research

A strength of this review is its integration of digital health, exercise prescription, rehabilitation, patient safety, ethics, and AI governance. It frames explainability and oversight as functions within patient-facing care pathways rather than only as technical or regulatory concepts.

Several limitations remain. This was a governance-oriented narrative review rather than a systematic review, scoping review, comprehensive evidence map, or meta-analysis, and it did not include formal risk-of-bias appraisal. Although the review reports search strings, dates, source counts, selection steps, source-role categories, and extraction fields, these elements are provided to make narrative source selection transparent rather than to claim exhaustive reproducibility or complete evidence mapping. Its findings are best interpreted as a conceptual synthesis rather than as conclusions from a fully reproducible systematic review.

The proposed framework and checklist remain to be tested prospectively through stakeholder co-design, patient-facing usability work, clinician workflow evaluation, implementation pilots, and safety studies. Future research can examine how explanation formats are understood, how review thresholds perform, how oversight affects workflow and cost, and how minimum safeguards can be implemented without increasing digital exclusion. As technical architectures, regulatory expectations, and digital care models evolve, periodic governance review is likely to remain necessary.

## 7. Conclusions

AI-generated exercise guidance may warrant safety-relevant governance attention when it shapes individualized exercise dose, progression, contraindication-sensitive decisions, or rehabilitation strategy. In these settings, generic model transparency alone is unlikely to be sufficient. Digital healthcare services can combine implementation-focused explainability, proportionate human oversight, escalation pathways, and post-deployment monitoring according to the embodied and clinical risks of the use case.

Systems that disclose evidence sources, explain recommendation logic, communicate warnings, present reasonable alternatives, and route higher-risk cases to professional review may support safer self-management and more accountable service delivery. These proposed considerations are best interpreted as conceptual implementation supports rather than validated instruments or formal regulatory classifications. Future work can test them through patient comprehension studies, clinician workflow studies, usability testing, implementation pilots, and safety evaluations.

## Figures and Tables

**Table 1:** Search, selection, data extraction, and synthesis summary for the narrative review.

Review Component	Revised Reporting
Search dates and sources	Initial searches were conducted on 10 January 2026 and updated on 15 May 2026 in PubMed/MEDLINE, Google Scholar, and official regulatory or guidance websites from China, the European Union, the World Health Organization, and the United States. During the present revision, a targeted journal-specific check of Healthcare was also conducted on 29 May 2026 to identify recent articles directly relevant to AI exercise prescription, patient-centered digital health decision support, rehabilitation, explainability, and equity.
Search strings	The PubMed/MEDLINE string combined AI terms (“large language model” OR “generative AI” OR “artificial intelligence” OR “explainable AI” OR “clinical decision support”), exercise and digital-health terms (“exercise prescription” OR “exercise guidance” OR “physical activity” OR “rehabilitation” OR “telerehabilitation” OR “digital health” OR “mobile health”), and governance/safety terms (“patient safety” OR “human oversight” OR “automation bias” OR “trust” OR “governance”). Google Scholar used equivalent phrase combinations, and official websites were searched with combinations of “artificial intelligence”, “generative AI”, “clinical decision support”, “medical device”, “transparency”, “human oversight”, “software”, “change control”, and “governance”. Google Scholar was used as a supplementary interdisciplinary source, with screening limited to the first 100 relevance-ranked results for each phrase combination to improve transparency.
Selection flow	Records or source documents identified: *n* = 207. Duplicate or overlapping records removed: *n* = 39. Records screened by title, abstract, or summary: *n* = 168. Records excluded at screening: *n* = 103. Full texts or full documents assessed: *n* = 65. Full-text or full-document exclusions: *n* = 28, including technical-only model papers (*n* = 7), diagnostic AI without transferable decision-support implications (*n* = 6), generic wellness or marketing sources (*n* = 5), insufficient exercise or patient-facing relevance (*n* = 6), and non-authoritative commentary (*n* = 4). Final included source set: *n* = 37, including three targeted Healthcare articles relevant to AI exercise prescription, patient-centered digital health decision support, disability-related rehabilitation, and implementation equity.
Eligibility and exclusion	Sources were included when they informed at least one operational domain: exercise safety, digital delivery, AI explainability, human oversight, regulatory governance, digital literacy, patient attitudes, or implementation accountability. Sources were excluded when they lacked healthcare implementation relevance, transferable decision-support implications, exercise or patient-facing relevance, or a clear evidentiary or policy basis.
Screening and disagreements	Screening and charting were conducted by two authors (K.P. and X.L.). Disagreements were discussed with C.H. until consensus was reached.
Data extraction	Extracted fields included source type, population or user group, clinical or service setting, AI system or digital health technology, exercise or rehabilitation context, safety concern, explainability mechanism, human oversight mechanism, governance implication, and relevance to patient-facing digital healthcare.
Synthesis and status of outputs	The synthesis used framework synthesis and governance mapping in four traceable steps: (1) charting source statements related to safety, explanation, oversight, and governance; (2) grouping recurrent concerns by evidentiary role and implementation context; (3) mapping those concerns to digital healthcare workflow points; and (4) translating the mapped considerations into the proposed implementation outputs after the literature-supported themes and selected policy signals had been synthesized. These outputs are proposed conceptual and implementation-oriented tools rather than validated checklists, scoring instruments, or regulatory classifications.
Evidence role and quality appraisal	No single risk-of-bias tool or systematic-review quality appraisal was applied because the review combined empirical studies, reviews, ethics papers, and policy or regulatory documents rather than a single systematic-review evidence corpus. Instead, sources were categorized by evidentiary role before synthesis: empirical studies and systematic reviews informed implementation and safety considerations; exercise-prescription and rehabilitation sources informed clinical plausibility; official documents informed governance signals; and conceptual papers informed interpretive concepts.
Reference verification	For the present revision, the full reference list was rebuilt from DOI-, PubMed-, publisher-, or official-source records; mismatched author, title, journal, year, volume, page, and DOI fields were corrected, and in-text citation placement was rechecked.

Note. Counts are reported to make the narrative source-selection process transparent. They are not intended to be interpreted as a PRISMA-style systematic review flow, exhaustive scoping review, or comprehensive evidence map, because the review was designed for conceptual and governance-oriented synthesis rather than exhaustive identification of all evidence or effect estimation.

**Table 2:** Thematic grouping of included sources and role in the narrative synthesis.

Thematic Source Group	Primary Role in the Narrative Synthesis	Representative Sources
Digital exercise delivery and exercise-prescription evidence	Informed why exercise guidance becomes safety-relevant when it specifies dose, progression, contraindication-sensitive action, or rehabilitation strategy, and supported attention to adherence, monitoring, and remote delivery.	[1,2,3,4,5,6,7,8,9,35]
Explainability, trust, and automation bias literature	Informed the distinction between generic transparency and reviewable explanation, including evidence sources, reasoning paths, automation bias, trust, and human-AI reliance.	[10,11,12,13,14,15,17,19,36]
Professional responsibility, ethics, and patient disclosure literature	Informed patient notification, informed use, professional responsibility, institutional trustworthiness, and the ability of clinicians and exercise professionals to question, revise, or reject AI output.	[13,16,18,27,31,32,33,35]
Regulatory and policy documents	Informed the selected governance signals related to intended use, medical-device or clinical-decision-support status, human oversight, lifecycle governance, change control, and public-facing generative-AI service obligations.	[20,21,22,23,24,25,26]
Digital literacy, patient/clinician attitudes, and equity literature	Informed implementation concerns related to user understanding, clinician acceptance, digital literacy, equity, and applicability in lower-resource or differently regulated settings.	[28,29,30,34,37]

**Table 3:** Selected regulatory and policy signals relevant to implementation governance.

Jurisdiction	Selected Source(s)	Key Regulatory Trigger	Service Implication
China	Interim Measures for the Management of Generative Artificial Intelligence Services (2023) [26]	Public-facing generative AI services are subject to baseline obligations concerning lawful, safe, and regulated deployment. The measure itself does not create a dedicated exercise-prescription test.	Supports upstream governance and service-provider accountability for app-based exercise guidance, but does not by itself define what point-of-use explanation is sufficient for individualized recommendations.
European Union	European Union Artificial Intelligence Act (Regulation (EU) 2024/1689) [21] and Medical Devices Regulation (EU) 2017/745 [22]	If an AI function is used as a medical device or safety component supporting therapeutic or rehabilitation decisions, the interaction between AI governance and medical-device regulation can move the system toward a higher-risk compliance pathway.	Strengthens risk classification, documentation, transparency, and human oversight, while service-level explanation formats remain important when exercise guidance is delivered through apps, wearables, or remote rehabilitation pathways.
United States	FDA Clinical Decision Support Software guidance (current 2026 version) [23], Good Machine Learning Practice guiding principles [24], and Predetermined Change Control Plan guidance [25]	Regulatory status turns on intended use, target user, device status, and whether a healthcare professional can independently review the basis for a recommendation; patient- or caregiver-facing functions may warrant closer scrutiny.	Highlights reviewability, labeling, lifecycle governance, and change control for digital healthcare services, while leaving implementation teams to define practical patient-facing explanation and escalation mechanisms.

**Table 4:** Practical explainability elements for AI-generated exercise guidance.

Explainability Element	Implementation Purpose	Practical Question	Example Service Feature
Evidence source disclosure	Supports justified reliance and source transparency	What is this recommendation based on?	Show a tappable evidence card with source, date, and evidence-grade badge, such as guideline, consensus statement, validated protocol, or curated rule set.
Risk-warning communication	Supports safer use and harm prevention	What could make this unsafe?	Use contraindication acknowledgement and display stop rules before high-intensity progression, symptom-sensitive training, or rehabilitation-sensitive tasks.
Reasoning-path clarification	Supports reviewability and clinical checking	Why was this recommendation selected?	Provide a concise rationale panel showing structured inputs, retrieved guidance, contraindication logic, monitoring feedback, and FITT-VP-aligned rules that shaped dose, progression, or modality.
Presentation of reasonable alternatives	Supports user choice and shared decision-making	What reasonable options are available?	Offer a lower-intensity, lower-impact, or professionally reviewed pathway with a brief explanation of why it was or was not prioritized.

**Table 5:** Simplified implementation checklist for AI-generated exercise guidance in digital healthcare.

Governance Area	Core Question	Minimum Safeguard	Example Action
Use-case triage	Is the function wellness support, personalized guidance, or safety-relevant decision support?	Classify risk before deployment using dose control, contraindication sensitivity, rehabilitation relevance, and likelihood of direct enactment.	Link each tier to review and documentation expectations.
Source transparency	Can users and reviewers see what the recommendation is based on?	Show evidence provenance, protocol source, structured-data basis, or rule-set basis.	Add source labels or concise evidence cards in patient and reviewer views.
Risk communication	What could make the guidance unsafe?	Display contraindications, stop rules, red flags, and escalation cues at the point of use.	Use warning acknowledgement before higher-risk progression.
Human review	Which outputs may warrant professional confirmation?	Define review triggers for high-risk, ambiguous, or vulnerability-sensitive cases.	Route flagged outputs to clinician or exercise-professional review.
Escalation pathways	What happens when symptoms worsen or risk increases?	Build referral or follow-up routes into the workflow.	Trigger contact, teleconsultation, referral, or temporary suspension.
Audit and monitoring	Can the service support review and improvement?	Retain logs of outputs, sources, versions, overrides, symptom reports, and escalation events where appropriate.	Use audit trails for quality review, incident analysis, and post-deployment monitoring.

Note. Items are proposed implementation considerations rather than validated mandatory standards, scoring criteria, or jurisdiction-specific regulatory standards, and can be adapted according to clinical context, user population, service capacity, and jurisdiction-specific regulations.

## Data Availability

No new datasets were generated or analyzed during this review. All source materials analyzed are publicly available and cited in the reference list.

## Abbreviations

AI, artificial intelligence; CDS, clinical decision support; EU, European Union; FDA, Food and Drug Administration; FITT-VP, frequency, intensity, time, type, volume, and progression; LLMs, large language models; mHealth, mobile health; US, United States.
